# The epigenetic memory of temperature during embryogenesis modifies the expression of bud burst-related genes in Norway spruce epitypes

**DOI:** 10.1007/s00425-017-2713-9

**Published:** 2017-06-02

**Authors:** Elena Carneros, Igor Yakovlev, Marcos Viejo, Jorunn E. Olsen, Carl Gunnar Fossdal

**Affiliations:** 1 Norwegian Institute of Bioeconomy Research, 1431 Ås, Norway; 2 Department of Life Sciences, University of Alcalá, Ctra. de Barcelona km 33.600, 28805 Alcalá De Henares, Madrid Spain; 3 Department of Plant Sciences, Faculty of Biosciences, Norwegian University of Life Sciences, 1432 Ås, Norway

**Keywords:** Bud phenology, Dehydrins, *EBB1* genes, Epigenetic memory, *FTL2*, *Picea abies*

## Abstract

**Epigenetic memory affects the timing of bud burst phenology and the expression of bud burst-related genes in genetically identical Norway spruce epitypes in a manner usually associated with ecotypes.**

In Norway spruce, a temperature-dependent epigenetic memory established during embryogenesis affects the timing of bud burst and bud set in a reproducible and predictable manner. We hypothesize that the clinal variation in these phenological traits, which is associated with adaptation to growth under frost-free conditions, has an epigenetic component. In Norway spruce, dehydrins (*DHNs*) have been associated with extreme frost tolerance. *DHN* transcript levels decrease gradually prior to flushing, a time when trees are highly sensitive to frost. Furthermore, *EARLY BUD BREAK 1* genes (*EBB1*) and the *FT*-*TFL1*-*LIKE 2*-gene (*PaFTL2)* were previously suggested to be implied in control of bud phenology. Here we report an analysis of transcript levels of 12 *DHNs*, *3 EBB1* genes and *FTL2* in epitypes of the same genotype generated at different epitype-inducing temperatures, before and during spring bud burst. Earlier flushing of epitypes originating from embryos developed at 18 °C as compared to 28 °C, was associated with differential expression of these genes between epitypes and between buds and last year’s needles. The majority of these genes showed significantly different expressions between epitypes in at least one time point. The general trend in *DHN* expression pattern in buds showed the expected reduction in transcript levels when approaching flushing, whereas, surprisingly, transcript levels peaked later in needles, mainly at the moment of bud burst. Collectively, our results demonstrate that the epigenetic memory of temperature during embryogenesis affects bud burst phenology and expression of the bud burst-related *DHN*, *EBB1* and *FTL2* genes in genetically identical Norway spruce epitypes.

**Electronic supplementary material:**

The online version of this article (doi:10.1007/s00425-017-2713-9) contains supplementary material, which is available to authorized users.

## Introduction

Changes in global climate have been considered to represent a significant challenge for sufficiently rapid adaptation of species subjected to strong seasonal environmental stresses. Plants can cope with these stressful conditions and become more resistant to future exposures through an epigenetic memory mechanism. Epigenetic mechanisms that generate or remove epigenetic marks play an important role in plasticity responses to the environment and contribute to stress memory and adaptation in plants (Sahu et al. 2013; Thellier and Lüttge 2013; Baulcombe and Dean 2014; Avramova 2015; Crisp et al. 2016). Epigenetic mechanisms involve covalent modifications of DNA and histones of the chromatin, affecting transcriptional activity by altering the regulation of gene expression. Therefore, epigenetic mechanisms could modulate the development, morphology and physiology of an organism, contributing to an adaptive capacity of species such as forest trees (Bräutigam et al. 2013; Pikaard and Mittelsten Scheid 2014). Stress-induced epigenetic modifications are reversible but can be mitotically and meiotically transmitted in the form of heritable epialleles (Iwasaki and Paszkowski 2014).

The existence of an epigenetic memory that regulates bud phenology and cold acclimation in Norway spruce is well documented in studies of plants resulting from zygotic embryogenesis indoors in greenhouses compared to outdoors (Bjørnstad 1981; Johnsen 1989a, b; Skrøppa et al. 2007, 2010; Yakovlev et al. 2010, 2012). Furthermore, the temperature and photoperiod conditions during zygotic and somatic embryogenesis epigenetically shift the growth cycle program of the embryos, giving rise to different epitypes from the same genotype (Yakovlev et al. 2014). Depending on the temperature sum experienced by the developing embryo, phenological events such as bud burst or bud set can be advanced or delayed in time. A warmer embryogenesis environment delays their onset compared to colder conditions (Johnsen et al. 1996; Hänninen et al. 2007; Kvaalen and Johnsen 2008). During embryogenesis 329 out of 735 genes encoding putative epigenetic regulators were shown to be differentially expressed (DEG) in different epitype-inducing temperatures. The majority of these epigenetic regulator DEGs are related to DNA and histone methylation, the sRNA pathway and putative thermosensing and signaling genes (Yakovlev et al. 2016). These genes could be the main epigenetic regulators impacting chromatin status during formation of the epigenetic memory. In plants from epitype seeds the expression of siRNA pathways genes, miRNAs and phytochromes show significant DEG between epitypes (Johnsen et al. 2005; Yakovlev et al. 2010, 2011), but other transcriptional differences between such epitypes has not been surveyed.

Despite the increasing evidence for the epigenetic impact on bud burst phenology (Yakovlev et al. 2011; Bräutigam et al. 2013; Yordanov et al. 2014), generally relatively little is known about the control of dormancy release, bud burst and re-initiation of growth in trees. Studies on transcript profiling and gene expression during dormancy release and bud burst have been carried out in woody species such as *Picea* (Yakovlev et al. 2006; El Kayal et al. 2011; Busov et al. 2016), *Populus* (Rohde et al. 2007; Yordanov et al. 2014), *Quercus* (Derory et al. 2006; Uneo et al. 2013), *Prunus* (Basset et al. 2006; Yamane et al. 2008), *Malus* (Wisniewski et al. 2015) and *Pyrus* (Tuan et al. 2016). During dormancy there is a temporary suspension of morphogenetic activity. After dormancy release, bud burst marks the onset of the growing season, a time when trees are susceptible to environmental stressors such as late spring/early summer frosts. Onset of growth is water demanding for rehydration of meristems, cell expansion and metabolic pathway recovery, thus water stress-related proteins are likely important in the bud burst processes. Dehydrins (DHNs) are hydrophilic members of the late embryogenesis abundant class of proteins and are described as putative dehydration protective proteins. In trees, high levels of DHNs have been associated with tolerance to freezing temperatures, winter dormancy and protection against water stress (Basset et al. 2006; Yakovlev et al. 2008; Perdiguero et al. 2012; Eldhuset et al. 2013; Strimbeck et al. 2015). Due to their physicochemical properties, DHNs are considered to play a protective role as stabilizers of nuclear or cytoplasmic macromolecules and membranes under conditions of low water availability (Campbell and Close 1997; Danyluk et al. 1998; Koag et al. 2003). Rinne et al. (1999) suggested that DHNs might create local pools of water molecules that sustain the metabolic processes crucial during stress and re-growth. Water-binding capacity of DHNs may account for their role in protecting enzymes during cold stress (Rinne et al. 1999; Graether and Boddington 2014) and decreasing the damages created by ice crystal formation (Wisniewski et al. 1999). Their water-binding ability may also promote vitrification or act to prevent membrane–membrane interactions to stop or counteract the loss of water (Strimbeck et al. 2015).

Besides *DHNs*, other genes have been described to affect bud phenology. In woody perennials, a putative APETALA2/Ethylene responsive transcription factor named EARLY BUD-BREAK 1 (EBB1) was suggested to regulate the re-initiation of shoot growth after winter dormancy, since transcript levels increase prior to and during bud burst (Yordanov et al. 2014; Wisniewski et al. 2015; Busov et al. 2016; Tuan et al. 2016). EBB1 has been suggested to be involved in the shoot apical meristem activation via stimulation of cell proliferation (Yordanov et al. 2014).

Modulation of tree growth in response to seasonal changes in temperature, day-length and light quality is controlled genetically, sharing common traits with control of flowering (Gyllestrand et al. 2007; Olsen 2010; Asante et al. 2011; Olsen and Lee 2011). In *Populus*, short day-induced bud formation is closely linked to a decreased expression of *FLOWERING LOCUS T* (*FT*) (Böhlenius et al. 2006). Norway spruce lacks such an *FT* gene (Nystedt et al. 2013), but contains an *FT*-*like* gene (*FTL2*) with similarity also to *TERMINAL FLOWER 1* (*TFL1*) in *Arabidopsis thaliana. FTL2* shows substantially increased expression in short days and light quality conditions resulting in bud set, indicating a critical involvement in inhibiting growth and induction of bud set, as verified in Norway spruce plants overexpressing the gene (Gyllestrand et al. 2007; Asante et al. 2011; Karlgren et al. 2011, 2013; Opseth et al. 2015).

The aim of the study was to examine if the epigenetic memory of temperature during embryogenesis impacts on the bud burst-related *DHNs*, *EBB1* and *FTL2* genes in Norway spruce by examining their differential expression profiles before and during bud burst in spring in buds and last year’s needles in different epitypes.

## Materials and methods

### Plant materials and sample collection

Samples for studying gene expression during bud burst initiation were collected from a single genotype (ID#A2K) of the full-sib family of *Picea abies* (L.) Karst. arising from cross ♀#2650 × ♂#2707. This genotype was generated by somatic embryogenesis, where the embryogenic cultures were subjected to temperature treatments known to induce the formation of different epitypes from the same genotype in a predictable and reproducible manner (Kvaalen and Johnsen 2008). Two epitypes were used, originating from “cold” embryogenesis environment at 18 °C (C-epitype; CE) and “warm” embryogenesis environment at 28 °C (W-epitype; WE), respectively. For each epitype, terminal branches (nearly 15 cm long) with the terminal bud and needles of the previous year were collected at the Norwegian Institute for Bioeconomy Research field trial (Hoxmark, Ås-Norway; 59º40′07,5N/10º43′07,7E) and immediately frozen in liquid nitrogen and stored at −80 °C until use. Four biological replicates from each of the different clones (four individual trees per clone) were collected per epitype. Samples were collected weekly from the same trees in 2011 at six time points from April 20th until May 25th, covering the bud burst time point for both epitypes. The plants were also inspected weekly for their bud burst status.

### RNA extraction

To study the dynamics of gene expression at different time points in the different epitypes a total of 96 samples, i.e. 48 bud and 48 needle samples, were processed separately. The rationale for studying needles in addition to buds was that leaves like shoot tips perceive environmental signals and that signaling from needles to buds in dormancy-related processes cannot be excluded (Wareing 1954; Thomas and Vince-Prue 1997). Also, to survive the winter, like shoot tips, needles have to be cold hardened and will de-harden in the spring before bud burst occur. The needles surrounding the terminal buds were removed to avoid interference with needles of the previous year. For tissue disruption a tissue lyser (RETCH MM300) bead mill was used, and RNA was purified using Epicentre MasterPure™ Plant RNA Purification Kit (Epicentre, Madison, WI, USA, #MPR09100) according to the manufacturer’s instructions. Contaminating DNA was removed from the total RNA samples using the above-mentioned kit, according to the supplier’s protocol. The total RNA preparations were stored at −80 °C until gene expression analyses. The quantity of total RNA was assessed by a NanoDrop 2000 Spectrophotometer (Thermo Scientific).

### Genes searching and sequence analysis

*DHNs* were selected from a screened and annotated set of ESTs in a Norway spruce database from suppressive subtraction hybridization (SSH) cDNA libraries, and the *FTL2* gene used has accession number EF633467 (previously named *TFL1*) (Yakovlev et al. 2008; Asante et al. 2011). For the expression study of the three spruce *EBB1* orthologs we used the http://www.congenie.org functional genome resource for various tissues. These orthologs were shown to be highly expressed in vegetative buds (Busov et al. 2016).

### Reverse transcription quantitative real-time PCR (RT-qPCR) analysis

RNA extracted from the four independent biological replicates of terminal buds and needles was employed for cDNA synthesis and subsequent RT-qPCR analysis. First-strand cDNA was synthesized from 375 ng of total RNA in 50 μl reaction volume using TaqMan^®^ Reverse Transcription Reagents (Applied Biosystems, Carlsbad, CA, USA, #N8080234) according to the manufacturer’s procedure. RT-qPCR amplification was performed in a 10 μl reaction volume, using 2 μl of cDNA solution as template, 5 μl of 2X Fast^®^ SYBR Green Master Mix and 200 nM of each primer. Gene specific primers for 12 Norway spruce *DHNs*, 3 orthologs of *EBB1* genes (*PaEBB1.1*, *PaEBB1.2* and *PaEBB1.3*) and *PaFTL2* were designed with the Primer 3 software (Rozen and Skaletsky 2000) with default parameters and amendments according to the following criteria: melting temperature around 70 °C and product size between 80 and 150 bp (Table 1). Gene expression analyses were performed using the ViiA 7 Real-time PCR system (Applied Biosystems) with standard cycling parameters. For data analysis, the arithmetic mean of four different biological replicates was calculated and a no-template control was run for each primer pair. Target gene expression was normalized to the average of transcript levels of the spruce *ACTIN* (*PaACTIN*), *TRANSLATION INITIATION FACTOR*-*5*-*ALPHA* (*PaelF5α*) and *α*-*TUBULIN* (*Paα*-*TUB*). Quantification was performed using the ViiA 7 Software (Applied Biosystems).

**Table 1:** Primer sequences used for RT-qPCR analyses of twelve dehydrins, three *EARLY BUD*-*BREAK 1* orthologs and the *FLOWERING LOCUS T*-*LIKE* 2 transcripts and three reference genes. Sequences are listed in the 5′–3′ direction

Gene IDs	Accession no.^a^	Forward primer	Reverse primer	Product length (bp)
*PaDHN 1*	MA_95995g0010	GCGGCCTATGCGGCAAGAA	TCGACGAGCCCCGCCTTCTG	95
*PaDHN 2.2*	MA_187114g0010	CGCGGGCTGTTCGGTTTGTT	CAGCGGACGCAAGCAGAGGA	115
*PaDHN 4.3*	MA_8320994g0010	GCGGCGGACAGCATTCTTCG	TTGTTATCGTGGCCCGGAAGC	100
*PaDHN 6*	MA_747559g0010	TCCCGGAGGCCGGAACAAGT	CGAAAGCGACATGGAGAGGTAGCC	102
*PaDHN 9*	MA_2408574g0010	TCACGGTCAGCAGGGGCAAG	AACCGGAGCCGGAGCCATGT	101
*PaDHN 13*	MA_205576g0010	CCAGCTCAGAAGGCGGGGTTT	TGGCATCCAGGCAGCATCTC	116
*PaDHN 23*	MA_144878g0010	GACTACCAGGACCGCAGCCACA	GAGTCTGGCCTCCGGGAATCA	103
*PaDHN 24*	MA_12179g0010	CCCGGCTGTCTGGAATGCTC	CCGCCAAAACCCCTAGCAGAACA	90
*PaDHN 35*	MA_10428426g0010	GGACGAAGGAACGCAGGATGA	CTGGCATCCGGGCAGCTTCT	154
*PaDHN 39*	MA_86965g0010	CGAGGAGGATAAGGGCGGGAAT	TGCGTGGGTTGTAGCAGGTG	115
*PaDHN 40*	MA_10257300g0010	CGTGGCAGGAGCAGGCATCA	GAGCCGGAGCGCAAAGACCA	102
*PaDHN 41*	MA_10434136g0010	CCGCGAGAAGCCCGTCCATAC	CACCAGCAAGAACACCGGCTGA	98
*PaEBB1.1*	MA_27642g0010	TGGCTTCGACACAACTGATCCTACCA	TGTGGTTGATCTTGTGGCTGCTGT	114
*PaEBB1.2*	MA_30120g0010	CGCTCCTTCTTACTTTGGCTTCGAC	CGTGTTGCTGCTGCTGTAATTCTGG	119
*PaEBB1.3*	MA_77420g0010	CCACCTCGGGTTGTGGTGTTTGTC	GTCATAAGGCCAGTTGAGCCAATGC	88
*PaFTL2* *(Previously TFL1)*	EF633467	ATGTTGGAGGAGACGACTTG	GTGTTGGATCGCTTGGACTA	81
*Pa ACTIN*	AY961918/MA_10427661g0030	TGAGCTCCCTGATGGGCAGGTGA	TGGATACCAGCAGCTTCCATCCCAAT	105
*Pa αTub*	X57980/ MA_93486g0010	GGCATACCGGCAGCTCTTC	AAGTTGTTGGCGGCGTCTT	66
*Pa elF5α*	AY961932/ MA_103714g0010	GCCGATGCGGGAGCTTCCAA	TGCAGGGCCTGGCCTTAATGACG	88

^a^Accession no. based on Norway spruce genome sequence v.1. (http://congenie.org/)

### Statistical analysis

One-way ANOVA and Tukey’s test were done for buds and needles separately using IBM SPSS Statistics for Windows, Version 22.0 (Armonk, NY: IBM Corp). (Supplemental ANOVA File 1). Gene expression values were also analyzed with a principal component analysis (PCA) in R Statistical Environment (R Core Team 2017) core functions plus the package FactoMineR (Lê et al. 2008) after correcting the missing values with the package missMDA (Josse and Husson 2016).

## Results

### Timing of bud burst differs between genetically identical Norway spruce epitypes

**Fig. 1:**
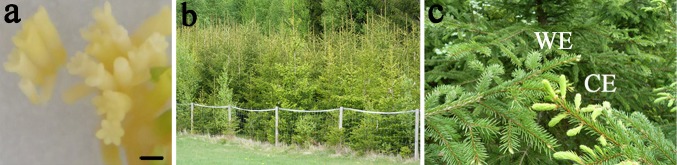
Epitypes of *Picea abies* **a** Somatic embryos cultured at 18 °C*. Bar* 1 mm. **b** Forest plantation of epitype trees generated from somatic embryos exposed to epitype-inducing temperature conditions. **c** Epitypes under identical spring conditions showing marked phenotypic differences in timing of bud burst-related to epigenetic memory of cold (CE; 18 °C) and warm (WE; 28 °C) temperature conditions during embryogenesis

**Fig. 2:**
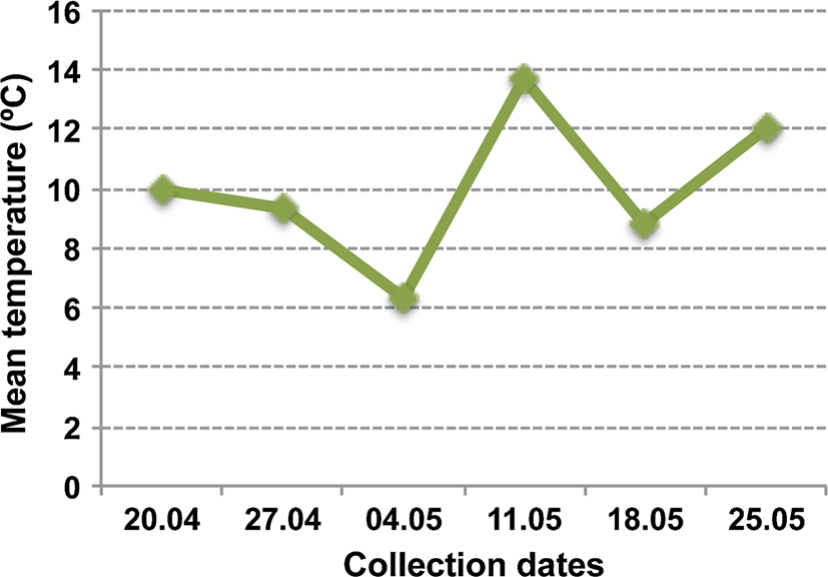
Mean temperatures registered at the different time points in spring in year 2011. CE and WE showed bud burst on May 11th and May 25th, respectively. Data were obtained from the Sørås Field Station for Agroclimatic studies, Norwegian University of Life Sciences, Ås, Norway

The epitypes generated from one single genotype examined in this work, were generated by somatic embryogenesis at two epitype-inducing temperatures (Fig. 1) as shown by Kvaalen and Johnsen (2008). The epitypes showed reproducible and predictable phenotypic differences related to the timing of bud burst (Fig. 1c) and bud set (Kvaalen and Johnsen 2008). Plants originating from embryos developed at 18 °C (CE) showed advanced bud burst with up to 2 weeks compared to plants originating from a warm embryogenic environment at 28 °C (WE) (Fig. 1c). For CE and WE bud burst occurred on May 11th and 25th (2011), respectively, which correlated with the first and second higher mean temperature periods recorded that spring (Fig. 2).

### Gene expression profiles differ between epitypes and plant parts

**Fig. 3:**
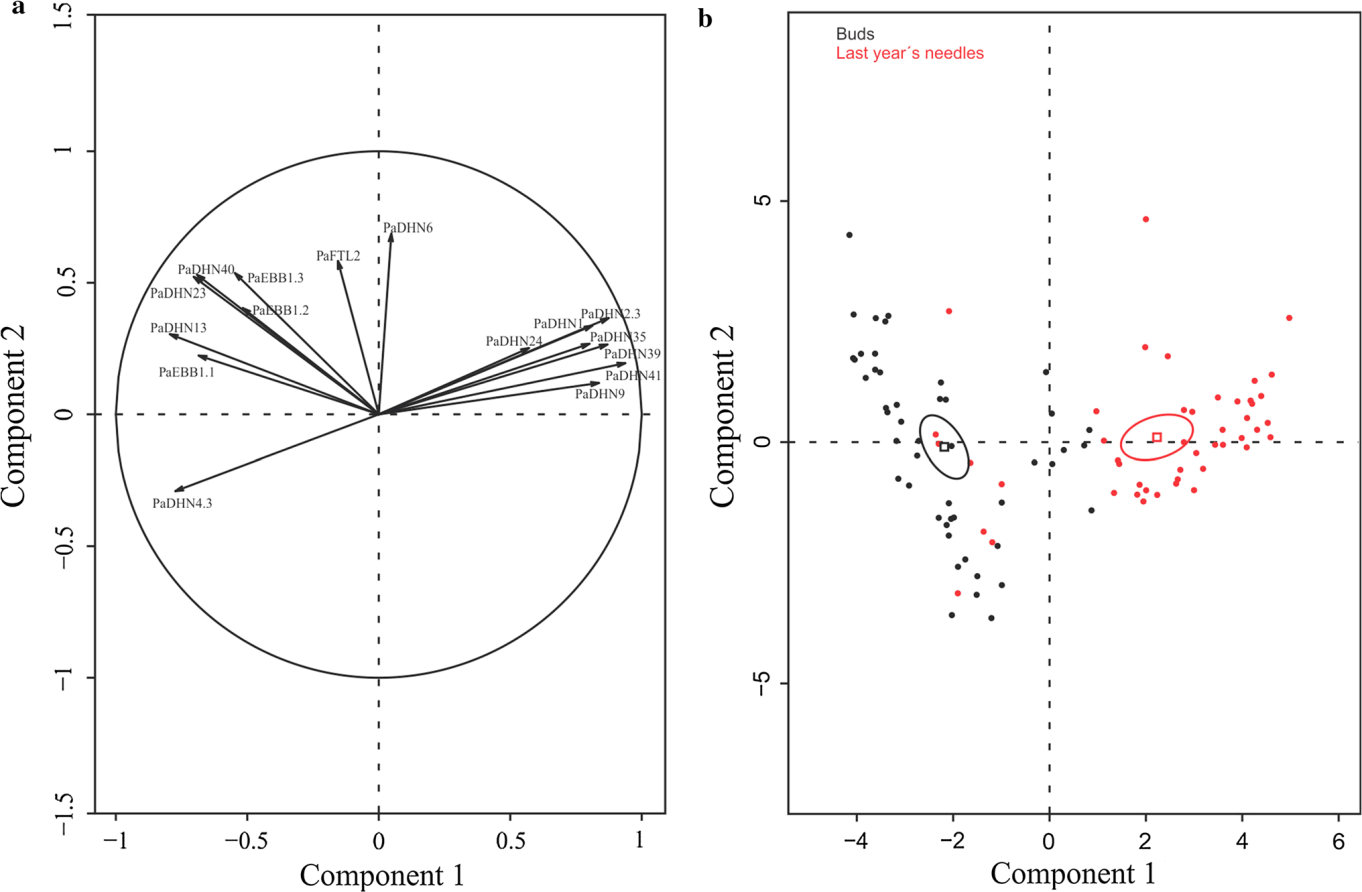
PCA factor maps for buds and last year’s needles from CE and WE epitypes of *Picea abies*. CE and WE trees originate from embryos formed under cold and warm epitype-inducing temperatures, respectively. Gene expression distribution (**a**) and sample distribution according to tissue type showing means (*squares*) and confidence ellipses (**b**). *Arrows* represent contribution intensity and direction of contribution

To study the overall pattern of gene expression and plant parts (buds and last year’s needles), PCA analyses were performed. In the analysis including both plant parts, the first component explained half of the variability and was significantly associated with plant part (*R* ^2^ = 0.6, probability value = 1.16e−20) (Fig. 3a, b). For this component, 14 out of the 16 genes studied showed significant correlation (Supplemental Table S1). The second component explained 15.94% of the variation with a weak but significant association with epitype (*R* ^2^ = 0.13, *P* value = 2.22e−4). No significant association was found among any of the components shown and sampling date (result not shown). Overall, two clear groups of *DHNs* could be identified: on one hand, *PaDHN 40*, *PaDHN 23*, *PaDHN 4.3* and *PaDHN 13,* and on the other hand, *PaDHN 41*, *PaDHN 39*, *PaDHN 35*, *PaDHN 2.2*, *PaDHN 24*, *PaDHN 1* and *PaDHN 9* (Fig. 3a).

**Fig. 4:**
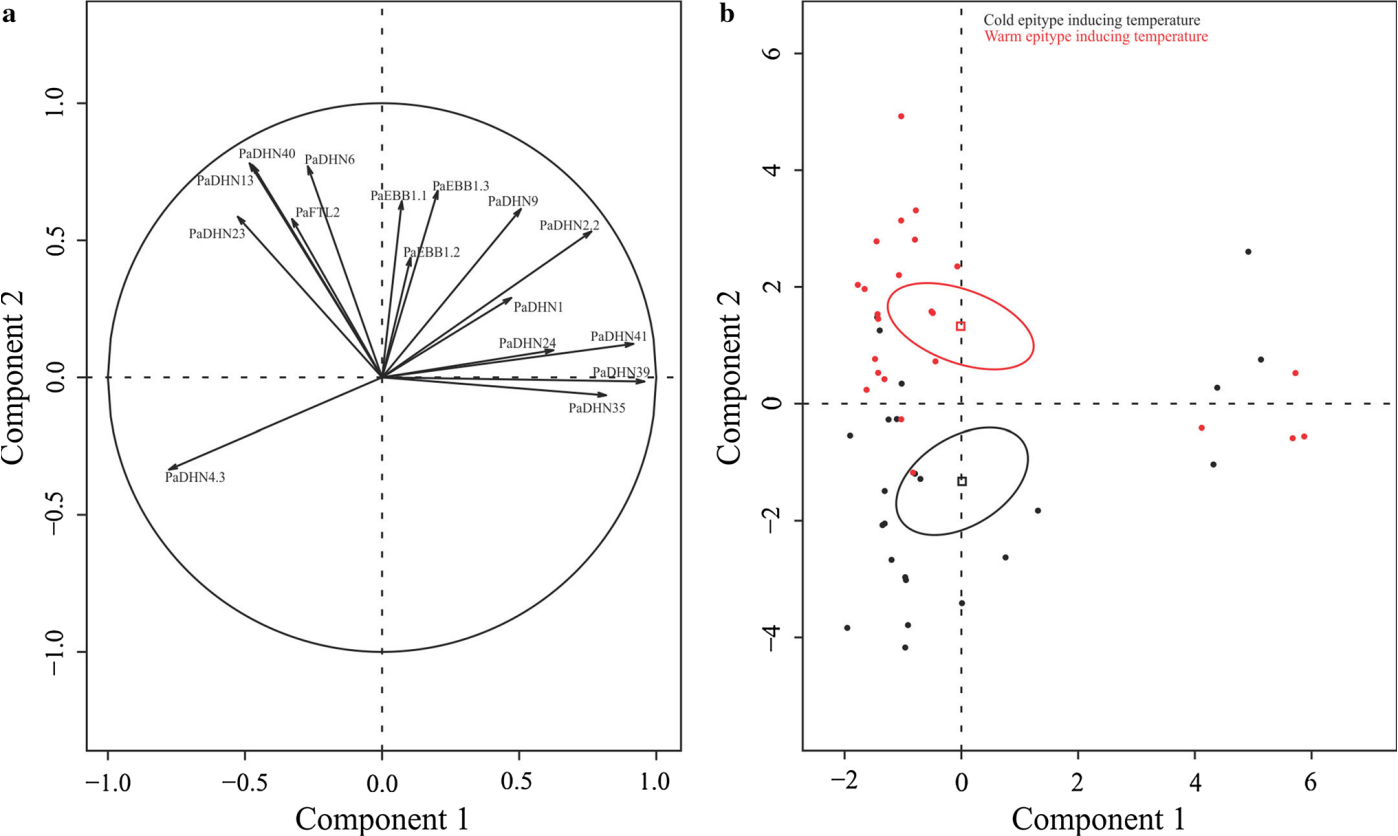
PCA factor maps for the buds of the CE and WE epitypes of *Picea abies*. CE and WE trees originate from embryos formed under cold and warm epitype-inducing temperatures, respectively. Gene expression distribution (**a**) and sample distribution showing means (*squares*) and confidence ellipses (**b**). *Arrows* represent contribution intensity and direction of contribution

**Fig. 5:**
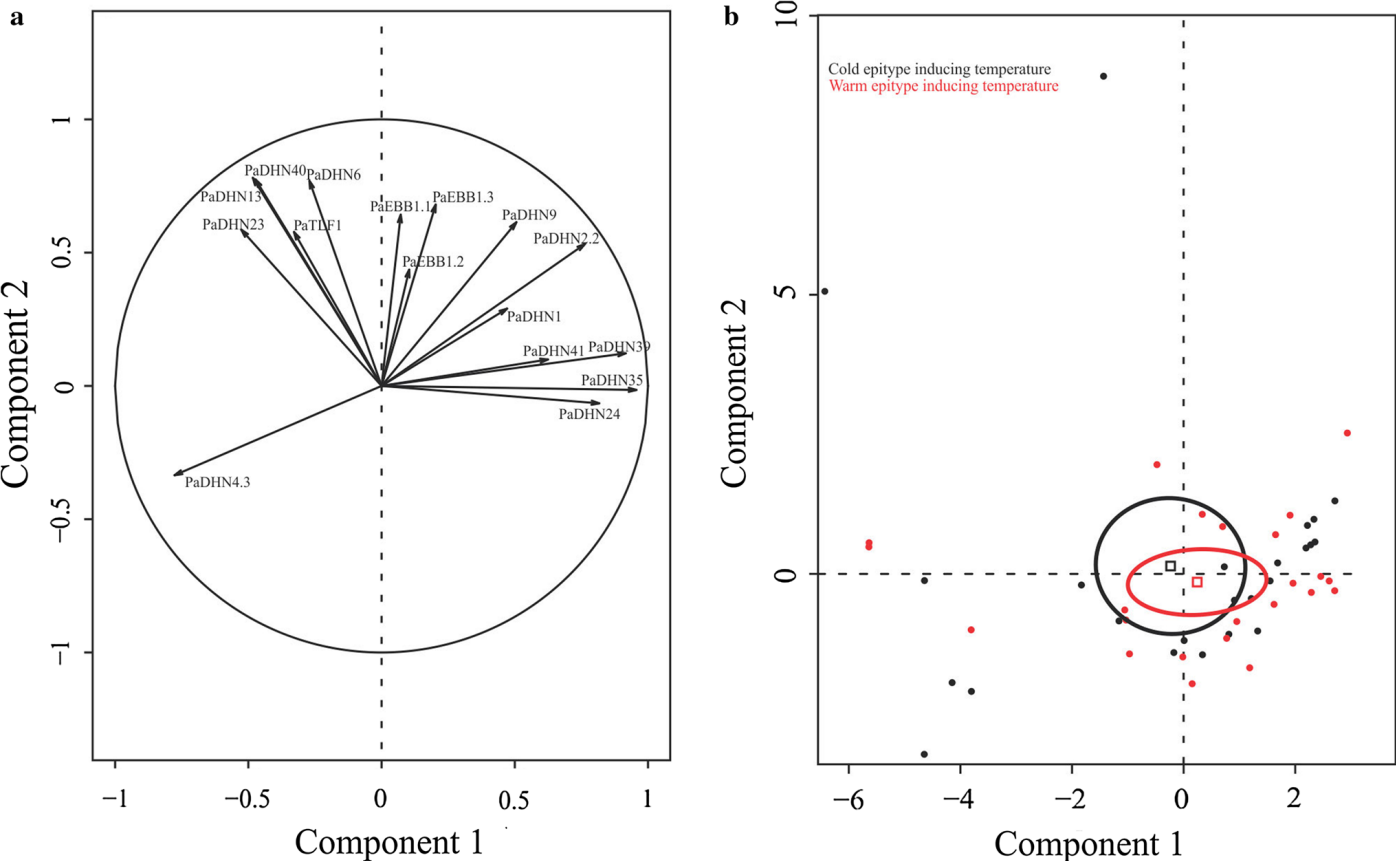
PCA factor maps for last year’s needles from CE and WE epitypes of *Picea abies*. CE and WE trees originate from embryos formed under cold and warm epitype-inducing temperatures, respectively. Gene expression distribution (**a**) and sample distribution according to tissue type showing means (*squares*) and confidence ellipses (**b**). *Arrows* represent contribution intensity and direction of contribution

Given the clear separation between buds and last year’s needles, PCA analyses were performed for each of the plant parts (Figs. 4 and 5) in order to get insight into the effects of the epitype-inducing temperature and the sampling date on gene expression. The *DHNs* maintained a similar grouping as described above (Figs. 4a, 5a). In the PCA for buds, the first component explained 34.05% of the variability and was mainly associated with the sampling date (*R* ^2^ = 0.93, *P* value = 1.45e−23; data not shown), with the 1st collection date being significantly different from the others (data not shown). The second component explained 27.69% of the variability and was significantly associated with the epitype-inducing temperature (Fig. 4b), (*R* ^2^ = 0.39, *P* value = 1.59e−06).

For last year’s needles, component one explained 39.7% of the variability and the second 22.19% (Fig. 5a). In contrast to buds, there was no significant association of epitype with any of the components (Fig. 5b). Nevertheless, the majority of the genes showed significant correlation with both components (Supplemental Table S2). The sampling date was significantly associated with component one (*R* ^2^ = 0.64, *P* value 1.40e−08), and the variability for the 3rd and 4th collection dates was explained by the positive part of the component, while the variability for the 1st collection date was explained by the negative part. Moreover, sampling date was also significantly associated with component two (*R* ^2^ = 0.22, *P* value 0.04) and the 5th collection date was partially explained by the positive part.

### Differences in gene expression profiles between epitypes

To examine if the timing of the expression of 12 *DHNs*, 3 *EBB1* genes and *PaFTL2* differed between epitypes, RT-qPCR analyses of expression pattern of these genes were carried out separately in buds and last year’s needles.

**Fig. 6:**
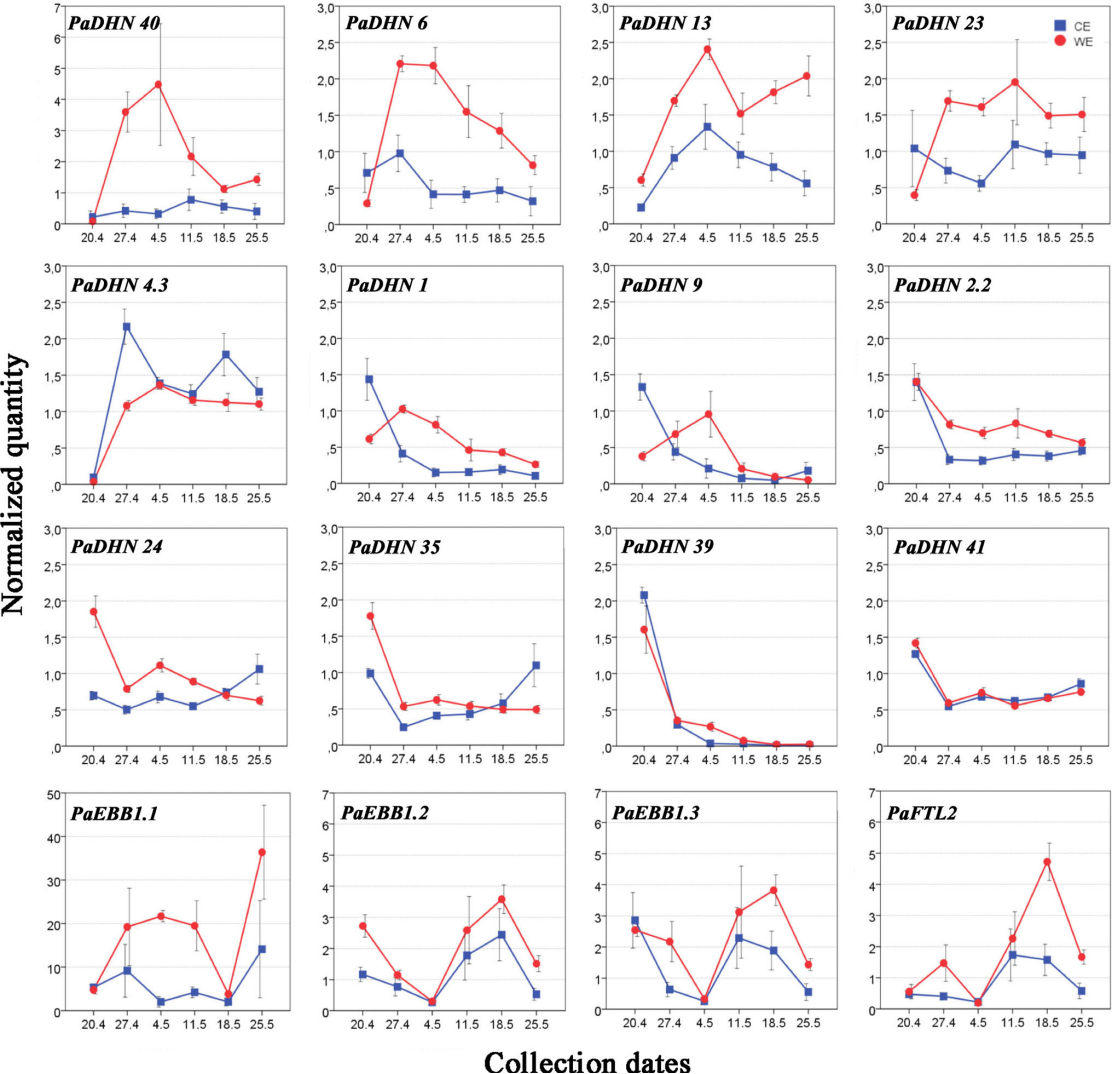
Expression profiles of the *Picea abies* dehydrins, the *EBB1* orthologs and the *FTL2* gene in terminal buds (or shoot tips after bud burst occurred) in CE and WE. For CE and WE, bud burst occurred on May 11th and 25th (2011), respectively. Data represent the arithmetic mean ± standard error of four different biological replicates at each sampling point. Quantified transcript level was normalized to the average of the spruce reference genes *PaACTIN*, *PaelF5α* and *Paα*-*TUB*

In buds, the majority of the *DHN* genes (*PaDHN 40*, *PaDHN 6*, *PaDHN 13*, *PaDHN 23*, *PaDHN 1*, *PaDHN 2.2*, *PaDHN 24*, *PaDHN 35* and *PaDHN 39*) showed differences in expression between the epitypes at several time points (mainly at 2nd and 3rd collection dates: 27th of April and 4th of May). Overall, the expression of these *PaDHNs* was significantly higher in the WE, consistent with its delayed bud burst compared to CE. Furthermore, *DHNs* could be grouped according to their expression patterns within CE or WE. For WE, the *DHN* genes *PaDHN 40*, *PaDHN 6* and *PaDHN 1* showed an increasing expression pattern up to 2nd and/or 3rd collection dates (27th of April and 4th of May), followed by a decreasing transcript level (Fig. 6). Even though no significant differences were observed for *PaDHN* 9 when comparing 1st and 2nd collection dates, it showed a similar expression pattern. *PaDHN 40* showed the highest transcript level for this epitype 3 weeks before bud burst (4th of May), reaching levels twofold higher than *PaDHN 6* at this time point. In WE, *PaDHN 13* had the same pattern as described above but at bud burst (25th of May) it showed increased expression. In this epitype, *PaDHN 23* and *PaDHN 4.3*, showed increasing expression up to four and 3 weeks before bud burst, respectively (27th of April and 4th of May), and then was maintained at quite stable levels during the sampling period (Fig. 6). In WE, *PaDHN 2.2*, *PaDHN 24*, *PaDHN 35*, *PaDHN 39* and *PaDHN 41* followed a decreasing trend towards bud burst as compared to the first sampling point (Fig. 6). Furthermore, in CE, an increasing expression of *PaDHN 13* and *PaDHN 4.3* was detected up to 1 or 2 weeks before bud burst (4th of May and 27th of April), followed by a decreasing transcript level (Fig. 6). Within the period studied there seemed to be a small increase in transcript levels for *PaDHN 24* and *PaDHN 35* after bud burst. In CE, *PaDHN 1*, *PaDHN 9*, *PaDHN 2.2*, *PaDHN 39* and *PaDHN 41* showed decreasing expression pattern towards bud burst (Fig. 6). Finally, several *DHNs* such as *PaDHN 40, PaDHN 6* and *PaDHN 23* showed more stable transcript levels in CE than in WE, as no significant differences in transcript levels were detected along the sampling period.

*EBB1* genes and *PaFTL2* showed differences in expression between the epitypes at several time points with overall significant higher expression in the WE epitype. For this epitype differences were observed for *PaEBB1.1* three and 2 weeks before bud burst (4th and 11th of May), and for *PaEBB1.2*, *PaEBB1.3* and *PaFTL2* at bud burst (25th of May) (Fig. 6). The highest transcript level was detected for *PaEBB1.1*, with a significant increment at WE flushing compared to earlier time points. CE and WE showed similar expression patterns with ups and downs for *PaEBB1.2*, *PaEBB1.3* and *PaFTL2*. No differential expression between CE and WE was detected for these genes at the time point corresponding to 1 week before bud burst for CE and 3 weeks before bud burst for WE (4th of May). Thereafter, their expression increased for the next 2 weeks and decreased drastically 2 weeks after flushing for CE and at bud burst for WE (25th of May) (Fig. 6).

**Fig. 7:**
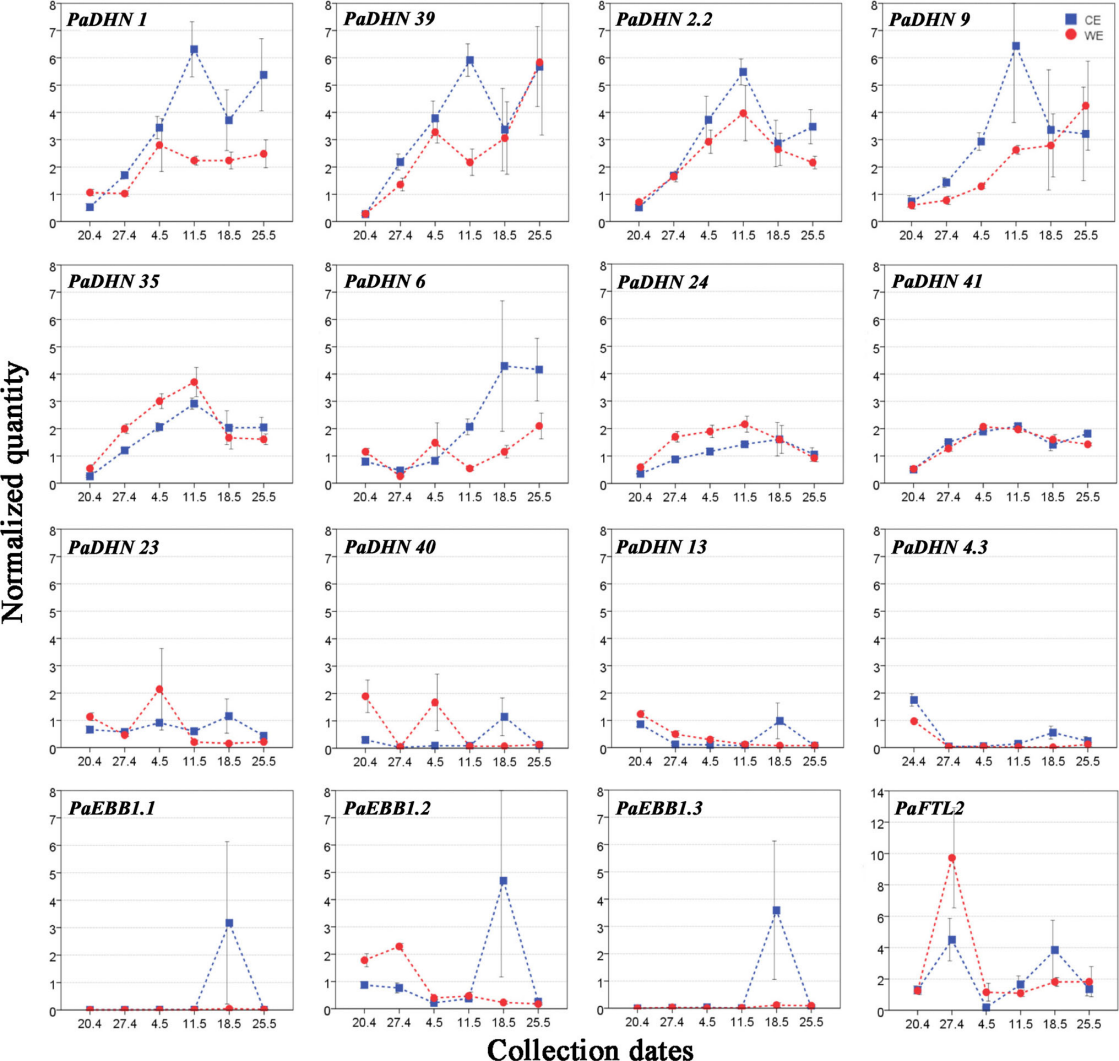
Expression profiles of the *Picea abies* dehydrins, the *EBB1* orthologs and the *FTL2* gene in last year’s needles of CE and WE. For CE and WE, bud burst occurred on May 11th and 25th (2011), respectively. Data represent the arithmetic mean ± standard error of four different biological replicates at each sampling point. Quantified transcript level was normalized to the average of the spruce reference genes *PaACTIN*, *PaelF5α* and *Paα*-*TUB*

In last year’s needles, the majority of the *DHN*s showed differences in expression between the epitypes at several time points, except for *PaDHN 2.2* where no significant differences were detected. For CE, *PaDHN 1*, *PaDHN 39* and *PaDHN 2.2* showed increasing gene expression up to bud burst with the highest transcript level at this time point (11th of May). This was followed by a decrease the week thereafter before the levels of these transcripts again increased (Fig. 7). For this epitype, *PaDHN 9* and *PaDHN 35* showed similar patterns but did not increase towards the final time point (Fig. 7). Despite that these *DHNs* showed the highest transcript level at bud burst, the transcript level of *PaDHN 35* showed less increase compared to *PaDHN 1*, *PaDHN 39*, *PaDHN 2.2* and *PaDHN 9*, which increased about twice as much. Also, in CE, *PaDHN 6* showed a clear increasing trend during the sampling period (Fig. 7). However, for this epitype very low or barely detectable expression was observed for *PaDHN 23, PaDHN 40, PaDHN 13* and *PaDHN 4.3* although slightly higher expression was detected a week after bud burst (18th of May) (Fig. 7). For the WE epitype, *PaDHN 1*, *PaDHN 39*, *PaDHN 9* and *PaDHN 6* in last year’s needles showed overall increasing transcript levels towards bud burst (Fig. 7). In this epitype, an increasing transcript level was observed for *PaDHN 2.2* and *PaDHN 35* with the highest expression 2 weeks before bud burst (11th of May) followed by decreased expression (Fig. 7). A similar trend but with lower amplitude was detected for *PaDHN 24* and *PaDHN 41* for both epitypes. For WE, *PaDHN 13* and *PaDHN 4.3* showed low transcripts level at first collection date (20th of April) followed by decrease to very low or barely detectable levels. Even though no significant differences were found for *PaDHN 23* and *PaDHN 40,* they showed a similar trend but with slightly higher transcript levels 4 weeks before bud burst (4th of May) (Fig. 7).

In last year’s needles a significant difference was observed between epitypes for *PaEBB1.2* (three first collection dates). For the early-flushing CE, the *EBB1* genes showed no significant differences between collection dates, only a trend of higher expression the week after bud burst (Fig. 7). For the late-flushing WE, decreasing expression towards bud burst was observed for *PaEBB1.2* whereas *PaEBB1.1* and *PaEBB1.3* showed no differential expression (Fig. 7). The epitypes showed similar behavior for *PaFTL2* with the highest transcript levels at 27th of April (Fig. 7).

### Differences in gene expression profiles between plant parts within epitypes

When buds and last year’s needles were compared, the expression pattern of the studied genes showed similar behavior within each epitype (Supplemental Figs. S1 and S2). For *PaDHN 2.2, PaDHN 35, PaDHN 39*, *PaDHN 9, PaDHN 1, PaDHN 41* and *PaDHN 24* transcript levels were significantly higher in needles compared to terminal buds for CE as well as WE. *PaDHN 6* and *PaFTL2* also showed this difference for CE. On the contrary, *PaDHN 4.3, PaDHN 13* and *PaEBB1.1* transcript levels were significantly higher for buds in both epitypes. *PaEBB1.2* and *PaEBB1.3* were significantly higher in buds than needles for WE.

In relation to bud burst, independently of the epitype, an opposite expression pattern was observed between buds and needles. Approaching bud burst, expression of *DHNs* decreased in buds (e.g. *PaDHN 2.2*, *PaDHN 39, PaDHN 9, PaDHN 1, PaDHN 41, PaDHN 24, PaDHN 6, PaDHN 40*). However, in needles, transcript levels of *PaDHN 39, PaDHN 9, PaDHN 1*, and *PaDHN* 6 were low at early spring (Supplemental Figs. S1 and S2), showing the highest expression level mainly at bud burst. It is noteworthy that some *DHNs* (including *PaDHN 4.3, PaDHN 13*, *PaDHN* 23 and *PaDHN 40*), *EBB1* genes and *PaFTL2*, in CE needles showed a peak of expression at one specific time point (18th of May) corresponding to the week after bud burst (Supplemental Figs. S1 and S2).

## Discussion

The main aim of this work was to investigate how the epigenetic memory of temperature during embryogenesis regulates bud burst in genetically identical epitypes (CE and WE) of Norway spruce. The studied eight year-old epitypes showed differences in the timing of bud burst. Consistent with the previously reported marked phenotypic differences (i.e. differences in timing of bud set) related to the memory of temperature during somatic embryo development (Kvaalen and Johnsen 2008), the CE showed flushing of terminal buds about 2 weeks earlier than WE. It is worth to mention that the first higher mean temperature recorded that spring, corresponded to the moment when CE exhibited bud burst (May 11th 2011), and the second higher temperature period corresponded to WE bud burst (May 25th 2011) (Fig. 2). The results confirm the existence of an epigenetic memory mechanism in Norway spruce that operates during embryo development and adjusts the timing of bud burst in the progeny in accordance with the temperature conditions during embryogenesis. At the time of this study, the epitype trees were 8 years old, which reflects that the epigenetic memory effect on bud burst is long-lasting, having implications for long-term growth under field conditions as observed by Skrøppa et al. (2007).

Here we report for the first time that the epigenetic memory affects expression of *DHN* genes, *EBB1* genes and *PaFTL2* in Norway spruce epitypes in relation to the timing of bud burst. The patterns of *DHN* gene expression between the two epitypes were noticeably different. Out of the 12 *DHNs* selected, transcript levels of 9 of them were significantly higher in buds of the late-flushing WE (Fig. 6) as compared to the early-flushing CE. These results resembles previous results in Norway spruce family materials where transcript levels of *DHNs* remained considerably higher in late-flushing compared to early-flushing families in the spring (Yakovlev et al. 2008). It is noteworthy that the regulation of *DHNs* is under tight control in this species as variation in transcript levels of *DHNs* within each epitype in our study was relatively low, allowing detection of significant differences between the epitypes under natural field conditions. Differences between the two epitypes were also observed for *EBB1* genes and *PaFTL2* in buds with the highest transcript levels for the WE. PCA for buds including all genes confirmed the significant effect of the epitype-inducing temperature (Fig. 4; Supplemental Table S3). In last year’s needles, the expression differed significantly in epitypes at several time points for 11 out of the 12 *DHNs* and for *PaEBB1.2* (Fig. 7). Early-flushing CE showed higher transcript levels for seven of the *DHNs*. However, transcript levels were higher for late-flushing WE for four of the *DHNs* and for *PaEBB1.2*. These differences show that the expression of these genes is affected by the epigenetic memory in buds as well as in last year’s needles. However, in spite of these specific significant differences (ANOVA) for the last year’s needles, and in contrast to the situation for buds, the PCA for the needles, did not show any clear association between epitype and gene expression (Fig. 5; Supplemental Table S2). These results for the needles demonstrate absence of a differential global expression pattern for the analyzed genes between epitypes. This may suggest a lack of a clear role of last year’s needles in the differential timing of bud burst in the epitypes.

It is likely that the differential expression patterns between epitypes in response to the increasing spring temperature are due to specific chromatin modifications established during embryogenesis. A diverse range of environmental stresses can alter such epigenetic marks (Eichten et al. 2014). Of these, vernalization (defined as the acquisition of flowering competence by prolonged cold exposure) remains the best-understood environmentally responsive process impacted by epigenetic mechanisms. In this respect, epigenetic memory enables plants to remember their experience of winter conditions to flower the following spring. The floral repressor gene *FLOWERING LOCUS C* (*FLC*) in *Arabidopsis thaliana* is transcriptionally repressed by cold exposure (Baulcombe and Dean 2014) and repression is epigenetically maintained during subsequent development in warmer temperatures. Physiologically, bud burst is the final stage of a series of processes related to dormancy release and cold deacclimation. Like vernalization, dormancy release requires or is promoted by long-term exposure to low temperatures, and chilling restores the ability to grow but does not promote growth (Rohde and Bhalerao 2007). The physiological similarities between vernalization and dormancy release lead to the hypothesis that trees such as Norway spruce might employ a molecular mechanism analogous to vernalization regarding the establishment of an epigenetic memory, as Norway spruce epitypes remember the prolonged cold winter to bud burst the next spring.

An opposite behavior of the buds and last year’s needles was particularly clear for the genes selected, as also confirmed by the overall PCA analysis including both plant parts (Fig. 3; Supplemental Figs. S1 and S2). It was unexpected that the level of most *DHNs* was kept so high in last year’s needles (Supplemental Figs. S1 and S2). Differences between buds and needles were also observed for *EBB1* genes, showing higher transcript levels in buds in most cases. On the contrary, for *PaFTL2* needles generally had the highest transcript levels (See Figs. S1 and S2, in Supplemental). The expression pattern of most of the *DHNs* in buds was significantly decreased as bud burst was approached, whereas in needles transcript levels were low at early spring, when plants supposedly were still frost resistant, showing an increment of transcripts over time.

In Norway spruce, the timing of bud burst was shown to be associated with a high rate of net photosynthesis followed by decrease in amount of sugar and increase in starch compounds (Egger et al. 1996). Studies on metabolite profiling have been performed in Norway spruce during bud development (Lee et al. 2014; Dhuli et al. 2014). These authors assessed changes in metabolite profiles not only in sugars but also in other solutes such as ABA, antioxidants, flavonoids, terpenoids, amino acids and lipids, which were accumulated in cells during short days and frost, corroborating their cryoprotective properties. Dhuli et al. (2014) examined metabolite changes in buds and needles of Norway spruce and European silver fir during artificial forcing, observing higher levels of distinct carbohydrates in needles compared to buds. In evergreen conifers, carbohydrates and photosynthates from the previous year’s needles support shoot growth until new needles develop (Hansen and Beck 1994). Major changes in sugar metabolism occur in conifer needles during acclimation and deacclimation (Larcher 2003; Angelcheva et al. 2014). The differences in carbohydrate metabolism between buds and needles might possibly explain the differences in the regulation of *DHNs*, *EBB1* genes and *PaFTL2*. On the other hand, DHNs play a role in cold tolerance of plants by maintaining low local water content, protecting the tissues against frost damage (Wisniewski et al. 1999). In Norway spruce, transcript levels of *DHNs* decrease gradually during the period immediately preceding bud burst (Yakovlev et al. 2008; Asante et al. 2009) which could be explained by the stage of development of buds at this period. The internal vegetative bud development preceding bud burst in Norway spruce during the spring includes the growth of the primordial shoot and the swelling of buds (Sutinen et al. 2009, 2012; Viherä-Aarnio et al. 2014). Simultaneously, the water content of the buds increases as a result of the growth of vascular tissue and lower concentration of sugar, reducing the frost hardiness significantly (de Fäy et al. 2000; Luoranen et al. 2010). Mature needles are highly protected from freezing and the dehydrin levels in last year’s needles are in general higher and not down-regulated during the studied period such as in buds, while flushed buds have no tolerance to frost at all. It seems that down-regulation of the *DHNs* needed for protection from freezing is a prerequisite for start of cell division and growth in Norway spruce (Yakovlev et al. 2008; Asante et al. 2009). Thus, since last year’s needles are fully-grown and do not continue to elongate they do not need to down-regulate the *DHNs* and can maintain high levels of these protective proteins during the spring.

*EBB1* family genes seem to play an essential role in a conserved mechanism controlling bud break in perennial plants (Busov et al. 2016). In poplar, *EBB1* has been suggested to regulate the re-initiation of shoot growth after winter dormancy, and transcript levels are undetectable in buds during the majority of the dormancy period but appear prior to and during bud break (Yordanov et al. 2014). *EBB1* homologs are found to be associated with the timing of bud burst in apple, grape, spruce and pear (Wisniewski et al. 2015; Busov et al. 2016; Tuan et al. 2016). The dynamics of *EBB1* gene expression in buds in Norway spruce appears more complex than its angiosperm trees. In angiosperms, *EBB1* orthologs are down-regulated in dormant and up-regulated in actively growing apices. In our work, *PaEBB1.2* and *PaEBB1.3* transcript levels increased at bud burst in CE and towards bud burst in WE (Fig. 6) and reverted to low levels after bud burst in CE and at bud burst in WE. Differences in expression pattern between angiosperms and gymnosperms could be related to the difference in the biology of dormancy between these two groups.

A similar expression pattern to *PaEBB1.2* and *PaEBB1.3* was observed for *PaFTL2.* Our results show a peak of expression for this gene at bud burst for CE and 1 week before bud burst for the WE. *FTL2* was shown to be up-regulated under short days in Norway spruce, indicating a critical involvement in inhibiting growth and induction of bud set (Gyllestrand et al. 2007; Asante et al. 2011; Karlgren et al. 2013; Opseth et al. 2015). Our study did not include bud set, but demonstrates relatively high expression also around bud burst. According to Gyllestrand et al. (2007), buds from adult trees in natural stands confirmed low expression levels for *PaFT4*, renamed by Karlgren et al. (2011) as *PaFTL2*, at early stages of bud burst in spring but a small increase was observed when buds had completely burst. Furthermore, it has been described that high expression of *PaFTL2* is retained in needles during bud set until spring, when it drops to an intermediate level concurrent with increasing day-length and temperature (Gyllestrand et al. 2007; Karlgren et al. 2013). This observation is consistent with our results for both CE and WE, as low expression levels of this gene were detected in last year’s needles in late spring.

In conclusion, we have revealed important differences in bud phenology in epitypes formed in response to cold and warm epitype-inducing temperatures as a result of an epigenetic memory of temperature during embryogenesis. Of the 12 *DHNs* analyzed, 9 were found to have significantly different expression patterns in buds related to epitype-inducing temperatures during embryogenesis. Also the expression of *EBB1*-genes and *PaFTL2* is clearly affected by the epigenetic memory. The epigenetic memory mechanism apparently provides plasticity in climatic adaptation of Norway spruce that impacts the expression of these genes.

### *Author contribution statement*

CGF, IY and EC conceived and designed the research. CGF, IY and EC conducted the experiments. EC ran RT-qPCRs and performed ANOVAs and Tukey’s tests. MV performed PCA analysis. EC and CGF wrote the manuscript with assistance from IY, MV and JEO. All authors read and approved the final manuscript.

## Electronic supplementary material

Below is the link to the electronic supplementary material.

Supplementary material 1 (DOCX 29 kb)

Supplementary material 2 (XLSX 399 kb)

Supplementary material 3 (DOCX 2351 kb)

## Abbreviations

CE: Cold embryogenesis environment (18 °C)
DHNs: Dehydrins
*EBB1*: *EARLY BUD BREAK 1*-genes
*FTL2*: *FT*-*TFL1*-*LIKE 2*-gene
PCA: Principal component analysis
WE: Warm embryogenesis environment (28 °C)
