# Supplementary material for: The epigenetic memory of temperature during embryogenesis modifies the expression of bud burst-related genes in Norway spruce epitypes
Source: Planta. 2017 Jun 2;246(3):553–66. doi: 10.1007/s00425-017-2713-9 (PMC5561168; doi:10.1007/s00425-017-2713-9)
Supplement: Supplementary file 1 — Supplementary material 1 (DOCX 29 kb) [file 425_2017_2713_MOESM1_ESM.docx]

**Table S1** PCA components´ summary for buds and last year´s needles of CE and WE epitypes of *Picea abies*. Genes, correlation with the two first components and *P* values are shown

| **Component 1** | | | **Component 2** | | |
| --- | --- | --- | --- | --- | --- |
| **Gene** | **Correlation** | ***P* value** | **Gene** | **Correlation** | ***P* value** |
| *PaDHN 41* | 0.93 | 2.53e-44 | *PaDHN 6* | 0.68 | 1.45e-14 |
| *PaDHN 2.2* | 0.87 | 6.51e-31 | *PaFTL2* | 0.58 | 5.88e-10 |
| *PaDHN 39* | 0.86 | 3.00e-30 | *PaEBB1.3* | 0.53 | 2.03e-08 |
| *PaDHN 9* | 0.83 | 4.14e-26 | *PaDHN 40* | 0.53 | 3.21e-08 |
| *PaDHN 1* | 0.81 | 1.72e-23 | *PaDHN 23* | 0.52 | 5.51e-08 |
| *PaDHN 35* | 0.80 | 1.90e-22 | *PaEBB1.2* | 0.40 | 4.89e-05 |
| *PaDHN 24* | 0.57 | 1.54e-09 | *PaDHN 2.2* | 0.36 | 2.67e-04 |
| *PaEBB1.2* | -0.51 | 8.00e-08 | *PaDHN 1* | 0.33 | 8.36e-04 |
| *PaEBB1.3* | -0.54 | 9.56e-09 | *PaDHN 13* | 0.30 | 2.69e-03 |
| *PaEBB1.1* | -0.68 | 1.96e-14 | *PaDHN 35* | 0.26 | 8.59e-03 |
| *PaDHN 40* | -0.69 | 8.89e-15 | *PaDHN 39* | 0.26 | 9.27e-03 |
| *PaDHN 23* | -0.70 | 1.80e-15 | *PaDHN 24* | 0.25 | 1.38e-02 |
| *PaDHN 4.3* | -0.77 | 3.89e-20 | *PaEBB1.1* | 0.22 | 2.95e-02 |
| *PaDHN 13* | -0.79 | 7.50e-22 | *PaDHN 4.3* | -0.29 | 4.26e-03 |

**Table S2** PCA components´ summary for last year´s needles of CE and WE epitypes of *Picea abies*. Genes, correlation with the two first components and *P* values are shown

| **Component 1** | | | **Component 2** | | |  |
| --- | --- | --- | --- | --- | --- | --- |
| **Gene** | **Correlation** | ***P* value** | **Gene** | **Correlation** | ***P* value** | |
| *PaDHN 41* | 0.91 | 3.10e-19 | *PaEBB1.3* | 0.93 | 9.93e-22 | |
| *PaDHN 2.2* | 0.80 | 6.13e-12 | *PaEBB1.1* | 0.81 | 2.76e-12 | |
| *PaDHN 9* | 0.79 | 3.91e-11 | *PaDHN 6* | 0.73 | 5.16e-09 | |
| *PaDHN 39* | 0.76 | 2.71e-10 | *PaDHN 40* | 0.45 | 1.22e-03 | |
| *PaDHN 1* | 0.65 | 4.55e-07 | *PaDHN 23* | 0.43 | 1.99e-03 | |
| *PaDHN 35* | 0.56 | 3.55e-05 | *PaDHN 2.2* | 0.41 | 3.33e-03 | |
| *PaDHN 24* | 0.54 | 6.99e-05 | *PaDHN 35* | 0.41 | 3.78e-03 | |
| *PaDHN 23* | -0.53 | 1.20e-04 | *PaDHN 1* | 0.37 | 8.93e-03 | |
| *PaDHN 40* | -0.63 | 1.74e-06 | *PaEBB1.2* | 0.37 | 1.01e-02 | |
| *PaEBB1.2* | -0.69 | 5.80e-08 | *PaDHN 39* | 0.36 | 1.06e-02 | |
| *PaDHN 4.3* | -0.77 | 2.38e-10 | *PaDHN 13* | 0.33 | 2.34e-02 | |
| *PaDHN 13* | -0.82 | 7.93e-13 | *PaFTL2* | 0.31 | 2.91e-02 | |

**Table S3** PCA components´ summary for the buds of CE and WE epitypes of *Picea abies*. Genes, correlation with the two first components and *P* values are shown

| **Component 1** | | | **Component 2** | | | |
| --- | --- | --- | --- | --- | --- | --- |
| **Gene** | **Correlation** | ***P* value** | **Gene** | **Correlation** | ***P* value** |  |
| *PaDHN 41* | 0.95 | 3.13e-26 | *PaDHN 23* | 0.78 | 5.94e-11 |  |
| *PaDHN 39* | 0.91 | 6.78e-20 | *PaDHN 40* | 0.77 | 1.07e-10 |  |
| *PaDHN 35* | 0.81 | 1.45e-12 | *PaDHN 6* | 0.76 | 1.64e-10 |  |
| *PaDHN 2.2* | 0.76 | 2.86e-10 | *PaEBB1.3* | 0.68 | 1.04e-07 |  |
| *PaDHN 24* | 0.62 | 2.10e-06 | *PaEBB1.1* | 0.64 | 8.62e-07 |  |
| *PaDHN 1* | 0.50 | 2.42e-04 | *PaDHN 1* | 0.61 | 3.42e-06 |  |
| *PaDHN 9* | 0.47 | 7.30e-04 | *PaDHN 13* | 0.58 | 1.21e-05 |  |
| *PaFTL2* | -0.32 | 2.26e-02 | *PaFTL2* | 0.57 | 1.72e-05 |  |
| *PaDHN 40* | -0.47 | 6.91e-04 | *PaDHN 2.2* | 0.53 | 9.88e-05 |  |
| *PaDHN 23* | -0.48 | 5.04e-04 | *PaEBB1.2* | 0.43 | 1.93e-03 |  |
| *PaDHN 13* | -0.52 | 1.22e-04 | *PaDHN 9* | 0.28 | 4.56e-02 |  |
| *PaDHN 4.3* | -0.77 | 8.61e-11 | *PaDHN 4.3* | 0.33 | 1.99e-02 |  |
