# Supplementary material for: The epigenetic memory of temperature during embryogenesis modifies the expression of bud burst-related genes in Norway spruce epitypes
Source: Planta. 2017 Jun 2;246(3):553–66. doi: 10.1007/s00425-017-2713-9 (PMC5561168; doi:10.1007/s00425-017-2713-9)
Supplement: Supplementary file 3 — Supplementary material 3 (DOCX 2351 kb) [file 425_2017_2713_MOESM3_ESM.docx]

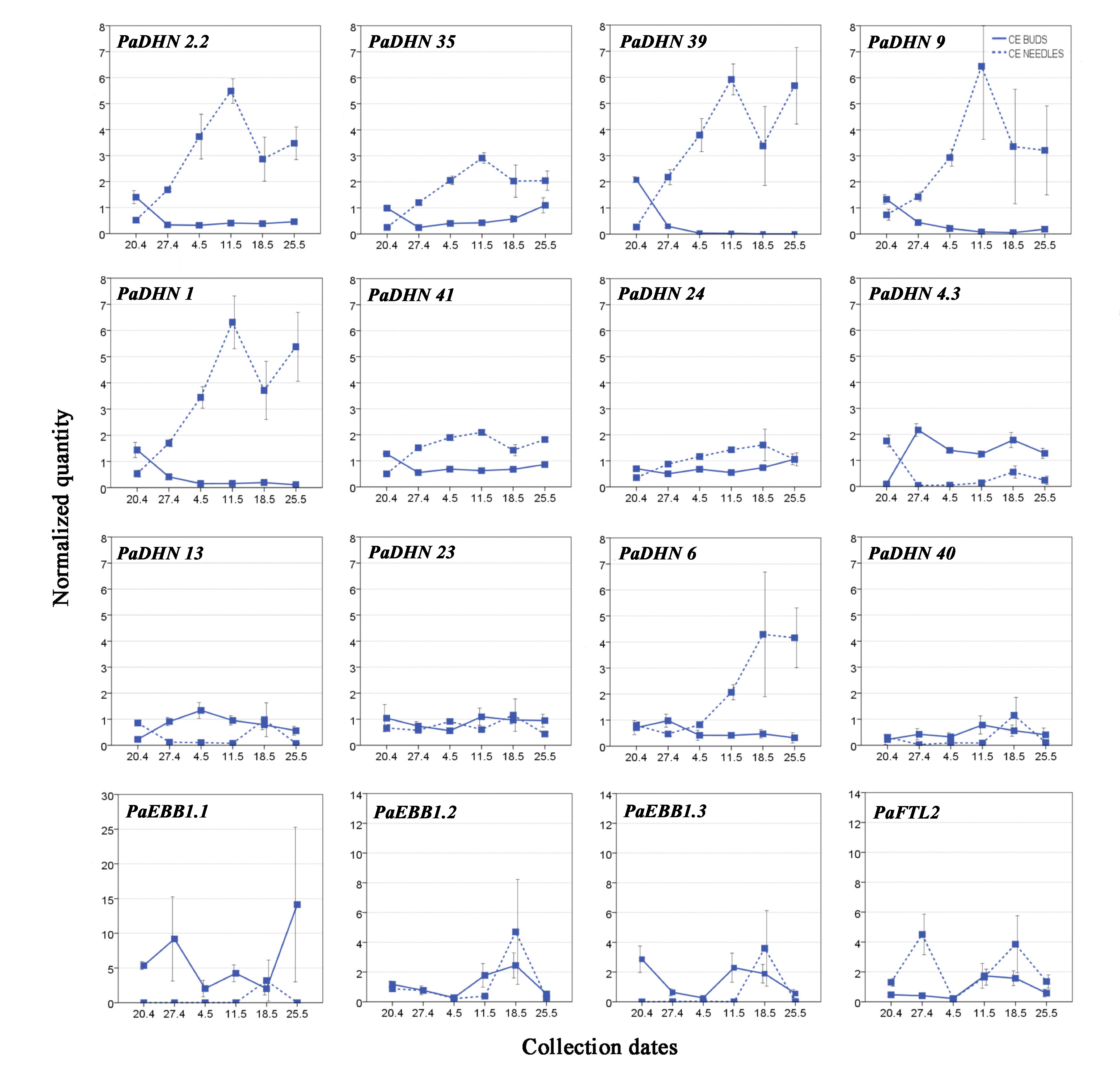


**Fig. S1:** Expression profiles of the *Picea abies* dehydrins, the *EBB1 orthologs* and the *FTL2* gene in terminal buds (or shoot tips after bud burst occurred) and last year´s needles of epitype originating from cold embryogenesis temperature (CE; 18ºC). For CE bud burst occurred May 11^th^ 2011. Data represents the arithmetic mean ± standard error of four different biological replicates at each sampling point. Quantified transcript level was normalized to the average of the spruce housekeeping genes *PaACTIN*, *PaelF5α* and *Paα-TUB.*

**
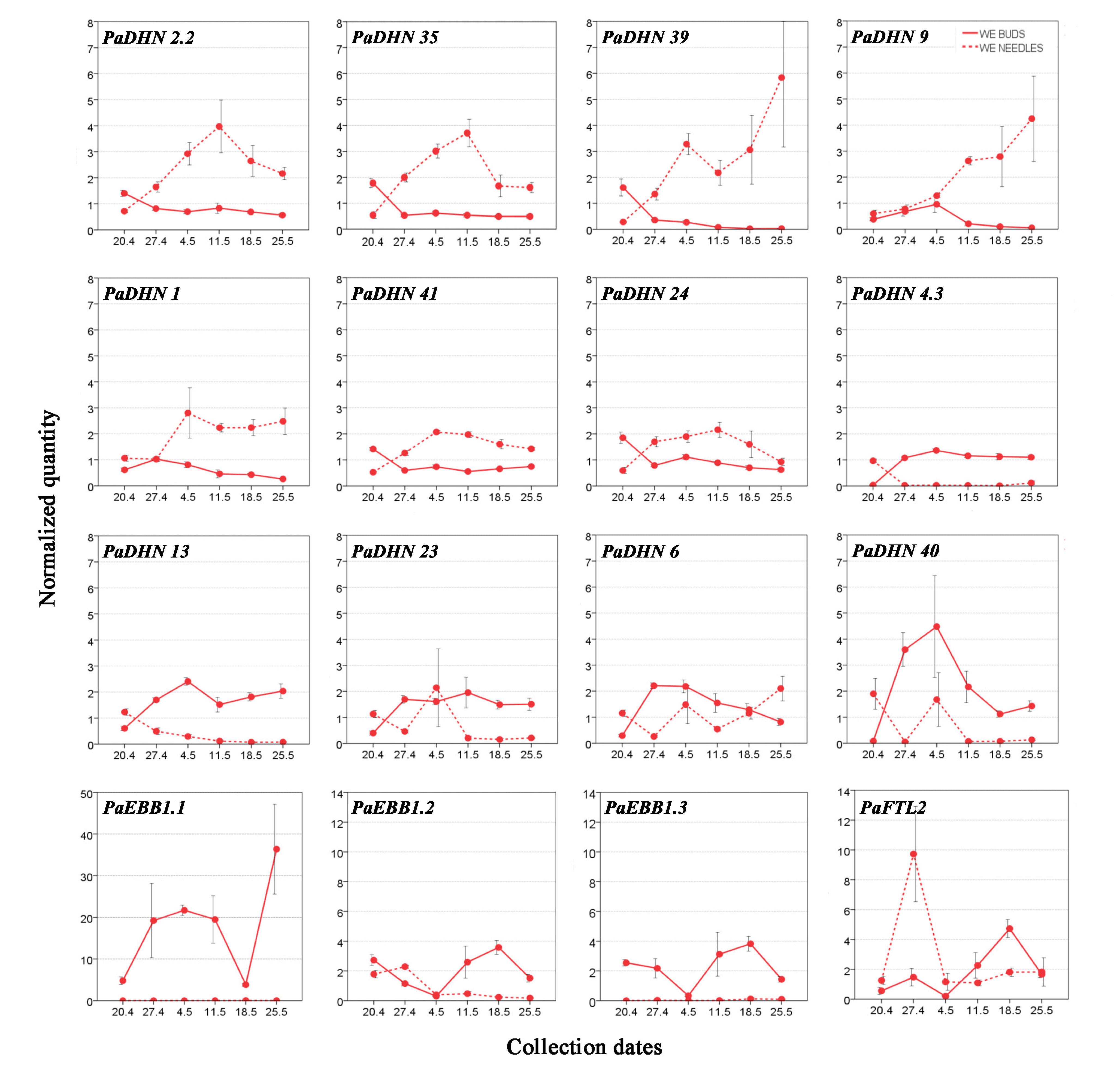
**

**Fig. S2:** Expression profiles of the *Picea abies* dehydrins, the *EBB1 orthologs* and the *FTL2* gene in terminal buds and last year´s needles of epitype originating from warm embryogenesis temperature (WE; 28ºC). For WE bud burst occurred May 25^th^ 2011. Data represents the arithmetic mean ± standard error of four different biological replicates at each sampling point. Quantified transcript level was normalized to the average of the spruce housekeeping genes *PaACTIN*, *PaelF5α* and *Paα-TUB.*
